# Six‐degrees‐of‐freedom pelvic bone monitoring on 2D kV intrafraction images to enable multi‐target tracking for locally advanced prostate cancer

**DOI:** 10.1002/mp.17465

**Published:** 2024-10-23

**Authors:** Emily A Hewson, Owen Dillon, Per R Poulsen, Jeremy T Booth, Paul J Keall

**Affiliations:** ^1^ Image X Institute Sydney School of Health Sciences The University of Sydney Sydney Australia; ^2^ Danish Centre for Particle Therapy Aarhus University Hospital Aarhus Denmark; ^3^ Northern Sydney Cancer Centre Royal North Shore Hospital Sydney Australia; ^4^ School of Physics The University of Sydney Sydney Australia

**Keywords:** intrafraction motion management, pelvic bone, six‐degrees‐of‐freedom motion, x‐ray image guidance

## Abstract

**Background:**

Patients with locally advanced prostate cancer require the prostate and pelvic lymph nodes to be irradiated simultaneously during radiation therapy treatment. However, relative motion between treatment targets decreases dosimetric conformity. Current treatment methods mitigate this error by having large treatment margins and often prioritize the prostate at patient setup at the cost of lymph node coverage.

**Purpose:**

Treatment accuracy can be improved through real‐time multi‐target adaptation which requires simultaneous motion monitoring of both the prostate and lymph node targets. This study developed and evaluated an intrafraction pelvic bone motion monitoring method as a surrogate for pelvic lymph node displacement to be combined with prostate motion monitoring to enable multi‐target six‐degrees‐of‐freedom (6DoF) tracking using 2D kV projections acquired during treatment.

**Material and methods:**

A method to monitor pelvic bone translation and rotation was developed and retrospectively applied to images from 20 patients treated in the TROG 15.01 Stereotactic Prostate Ablative Radiotherapy with Kilovoltage Intrafraction Monitoring (KIM) trial. The pelvic motion monitoring method performed template matching to calculate the 6DoF position of the pelvis from 2D kV images. The method first generated a library of digitally reconstructed radiographs (DRRs) for a range of imaging angles and pelvic rotations. The normalized 2D cross‐correlations were then calculated for each incoming kV image and a subset of DRRs and the DRR with the maximum correlation coefficient was used to estimate the pelvis translation and rotation. Translation of the pelvis in the unresolved direction was calculated using a 3D Gaussian probability estimation method. Prostate motion was measured using the KIM marker tracking method. The pelvic motion monitoring method was compared to the ground truth obtained from a 6DoF rigid registration of the CBCT and CT.

**Results:**

The geometric errors of the pelvic motion monitoring method demonstrated sub‐mm and sub‐degree accuracy and precision in the translational directions ($T_{\mathrm{LR}}$, $T_{\mathrm{SI}}$, $T_{\mathrm{AP}}$) and rotational directions ($R_{\mathrm{LR}}$, $R_{\mathrm{SI}}$, $R_{\mathrm{AP}}$). The 3D relative displacement between the prostate and pelvic bones exceeded 2, 3, 5, and 7 mm for approximately 66%, 44%, 12%, and 7% of the images.

**Conclusions:**

Accurate intrafraction pelvic bone motion monitoring in 6DoF was demonstrated on 2D kV images, providing a necessary tool for real‐time multi‐target motion‐adapted treatment.

## 1 INTRODUCTION

Treating patients with locally advanced or oligometastatic cancer using radiation therapy typically requires multiple targets to be irradiated simultaneously. However, treatment accuracy may be compromised due to differential motion between independently moving structures.^1^, ^2^ To compensate for relative motion between targets, large planning target volume (PTV) margins can be applied^3^, ^4^ to ensure each target receives the desired prescription dose, however, this is a suboptimal solution due to the resulting increase in dose to the healthy tissue.^5^

One patient subset requiring multi‐target treatment are those with locally advanced prostate cancer who can benefit from simultaneous prostate and pelvic node irradiation.^6^ While the prostate can undergo motion of up to 15 mm,^7^ this motion is largely uncorrelated with the pelvic nodes,^3^ which remain approximately fixed to the pelvic vasculature.^8^ Thus, when the patient is being aligned for radiation therapy treatment, a compromise must be made when aligning the radiation beam with the targets, and often the prostate is prioritized.^9^, ^10^ Decreases in dose coverage for the lymph nodes of 5%, 15%, and 25% have been seen when a patient is set up with 5, 10, and 15 mm prostate displacements respectively during intensity‐modulated radiation therapy treatment.^11^ In addition to translational displacements relative to the prostate, variations in pelvic rotation may also be present, leading to further setup errors. Despite the potential for dosimetric consequences resulting from geometric displacements of the pelvis, the six‐degrees‐of‐freedom (6DoF) position of the pelvis is generally not monitored and cannot be accounted for during standard radiation therapy treatment without compromising the primary target.

Treatment methods that account for interfraction displacements of the prostate and pelvic lymph nodes through online adaptation have been demonstrated.^12^, ^13^ However, online adaptive methods do not account for patient movement or internal prostate motion during treatment delivery which have shown considerable deviations in some patients.^7^, ^14^ To adapt to intrafraction motion of the targets, real‐time multi‐target MLC tracking has been proposed^15^, ^16^ which would require accurate intrafraction motion inputs for each target to be input.

Existing tumor motion tracking systems such as the CyberKnife^17^ and Radixact^18^ have been designed to track tumor motion during radiation therapy delivery, however, are limited to adapting to single targets. Alternatively, Kilovoltage Intrafraction Monitoring (KIM) uses hardware on a standard linear accelerator to monitor tumor motion in real time by estimating 6DoF motion as observed in intrafraction kV images.^19^, ^20^ KIM has previously been implemented in clinical trials to treat prostate^21^ and liver^22^ cancer patients, and has demonstrated sub‐mm and sub‐degree geometric accuracy for monitoring the motion of fiducial markers implanted in the prostate.^23^ So far, KIM has also been limited to monitoring the motion of a single target to guide motion adaptation.

The aim of this study was to enable multi‐target motion monitoring by developing and assessing a method to calculate the translation and rotation of the pelvic bone in intrafraction kV images as a surrogate for pelvic lymph node displacement. The resulting method could be integrated into a framework to enable simultaneous real‐time motion monitoring of both the prostate and pelvic lymph nodes and used to guide multi‐target treatment adaptation.

## 2 METHODS

A method to track the pelvic bone translation ($T_{\mathrm{LR}}$, $T_{\mathrm{SI}}$, $T_{\mathrm{AP}}$) and rotation ($R_{\mathrm{LR}}$, $R_{\mathrm{SI}}$, $R_{\mathrm{AP}}$) in intrafraction kV images were developed and described in detail below. The method was tested retrospectively on images collected from 20 prostate cancer patients who were treated in the Trans‐Tasman Radiation Oncology Group (TROG) 15.01 Stereotactic Prostate Ablative Radiotherapy with KIM (SPARK) trial.^21^ The anonymized patient images are publicly available.^24^

### 2.1 The multi‐target motion monitoring method

**FIGURE 1:**
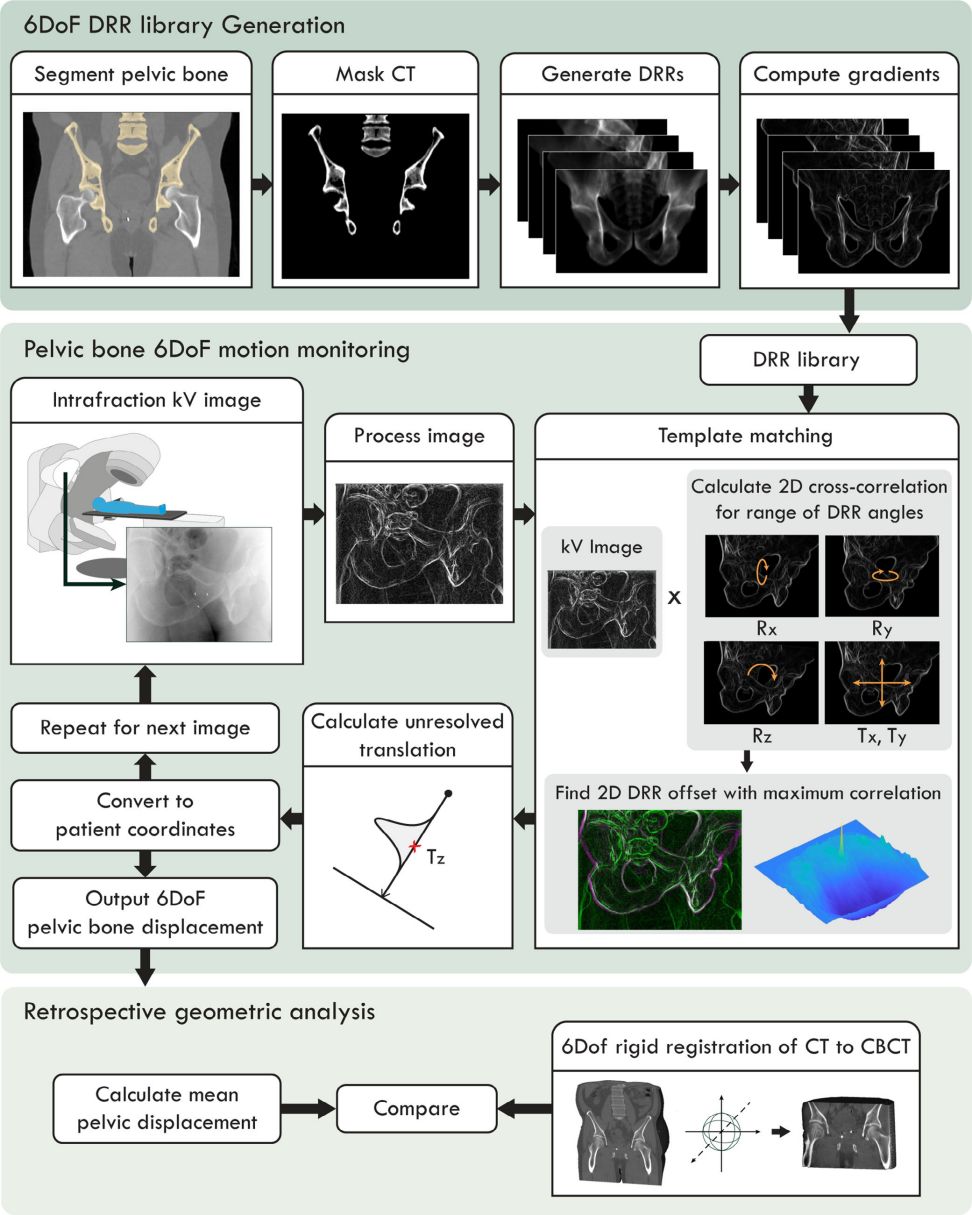
The workflow of the intrafraction six‐degrees‐of‐freedom pelvic motion monitoring method.

An outline of the multi‐target motion monitoring method is illustrated in Figure 1. The method performed template matching to identify the position of the pelvic bone in the kV projections, requiring a set of reference images, and is described in detail below.

#### 2.1.1 Pre‐treatment template generation

To generate a template of the anatomy of interest, the pelvic bone on each patient's planning CT was contoured and used to mask the CT volume. The femurs were excluded from the contour. Digitally reconstructed radiographs (DRRs) were generated of the masked CT volumes using the open‐source Reconstruction Toolkit (RTK).^25^ As the expected shape of the pelvic bone on the 2D treatment images would vary depending on the gantry angle, DRRs at a range of projection angles were generated to form a 6DoF library for each patient. DRRs were generated for angles around the superior‐inferior ($R_{\mathrm{SI}}$) axis ranging from 0° to 359.5° in increments of 0.5°. For each $R_{\mathrm{SI}}$ angle, projections with the pelvic bone rotating out of the imaging plane (*R_x_*) were also generated, ranging from ‐6° to +6° in increments of 0.5°. The magnitude of the gradients for each DRR in the *x* and *y* directions were then computed so that the edges of the bony anatomy would form the dominant feature in each template.

#### 2.1.2 6DoF motion monitoring of the pelvic bone

Pelvic bone segmentation was performed on kV images acquired with a known gantry angle. The method implemented three‐frame temporal averaging and a median filter to reduce image noise, followed by a Gaussian bandpass filter to enhance the edges of the bony anatomy in the image. The fiducial markers implanted in the prostate for prostate motion monitoring were automatically masked in the kV images. The gradients of each kV image were calculated to generate an image that highlighted the edges of the bony anatomy, consistent with the method used for template generation.

Template matching was performed by calculating the 2D normalized cross‐correlation of the template and the kV treatment image. The *x* and *y* values that corresponded to the maximum normalized cross‐correlation coefficient were used to determine the 2D displacement ($T_{x}$ and $T_{y}$) of the pelvic bone during treatment from the planning CT. $T_{x}$ and $T_{y}$ were computed at a resolution consistent with the acquired image resolution. The search region for $T_{x}$ and $T_{y}$ was limited to within 1 mm of the last measured position in each direction between image acquisitions. This calculation was repeated for a selection of DRRs from the generated library, to check rotations close to the expected rotation of the pelvis in the $R_{x}$ and $R_{y}$ directions. Each template image was also rotated around the imaging axis to calculate the normalized cross‐correlation for varying $R_{z}$ rotations. The DRR and $R_{z}$ rotation that had the highest normalized cross‐correlation value were used to determine $R_{x}$, $R_{y}$, and $R_{z}$. By finding the combination with the maximum normalized cross‐correlation coefficient, $T_{x}$, $T_{y}$, $R_{x}$, $R_{y}$, and $R_{z}$ were determined simultaneously. For the first treatment image, rotations ranging ± 6° from the planned pelvic bone position were tested, and for subsequent images rotations ranging ± 0.5° from the previous image were tested.

The final unresolved motion, translation along the imaging axis ($T_{z}$), was calculated using a probability‐based method previously described by Poulsen et al.^26^ This method assumes a 3D Gaussian probability density function (PDF) for the target position. An initial PDF was built using the position of the pelvis observed in the first 20 projections acquired using maximum likelihood estimation, and then updated with each incoming image. Then, the unknown position of the pelvis along the direction of the imager axis was estimated by finding the expectation value determined by the 1D Gaussian distribution along the imaging axis.

Finally, a rotational transformation was applied to determine the pelvic bone displacement in the patient coordinates ($T_{\mathrm{LR}}$, $T_{\mathrm{SI}}$, $T_{\mathrm{AP}}$ and $R_{\mathrm{LR}}$, $R_{\mathrm{SI}}$, $R_{\mathrm{AP}}$) with respect to the planning CT.

Prostate motion was monitored using KIM, which automatically segmented fiducial markers implanted in the prostate on the same kV image and estimated the 3D motion based on a 3D Gaussian PDF. Rotation of the prostate was then calculated using an iterative closest point algorithm for each marker to calculate a rotation matrix.^20^, ^27^

### 2.2 Retrospective geometric accuracy analysis

To evaluate the geometric accuracy of the pelvic tracking method described above, the method was tested retrospectively on prostate patient data. The patients included in this study were treated using a Varian TrueBeam linear accelerator, at three different treatment institutions. Two‐dimensional images acquired using the onboard kV imaging system on the linear accelerator were tested for 20 patients, who were each treated in five fractions. Each patient was treated using stereotactic body radiation therapy (SBRT) guided by KIM which provided real‐time 6DoF motion of the prostate.^23^

Due to the limited field of view of the intrafraction KIM images, the multi‐target motion monitoring method was tested on 2D kV projections acquired for the pre‐treatment cone‐beam computed tomography (CBCT) image at the beginning of each treatment to guide the initial patient set‐up. These images were acquired over a 200° arc at a rate of 14 Hz, with an average of 470 full‐fan projections acquired per CBCT. The projections had an average angle separation of 0.4°. The imaging system had a source‐to‐axis distance (SAD) of 1000 mm and a source‐to‐detector distance (SDD) of 1500 mm. Each image was acquired with a resolution of 1024 × 774 pixels and a pixel size of 0.388 × 0.388 mm^2^, and then cropped to a field size of 180 × 180 mm^2^ for pelvic motion monitoring.

The ground truth offset between the pelvic bone displacement during CBCT acquisition and in the planning CT was evaluated by performing an automatic 6DoF rigid registration of the planning CT to the 3D reconstructed CBCT using the Elastix toolbox.^28^ To ensure that the volumes were registered to the bony anatomy, the CT images were masked with the pelvic bone segmentations and registered to each CBCT. The planning CTs were acquired with 1.5, 2, and 2.5 mm slice thicknesses depending on the treatment institution. Both the translational and rotational displacements were calculated and compared to the output of the pelvic motion tracking method. The rationale for choosing this ground truth is that the mean of the real‐time 6DoF tracking during the image acquisition should equal the reconstructed image registration, assuming that the means from these two processes should be similar.

## 3 RESULTS

### 3.1 6DoF pelvic motion monitoring accuracy

**FIGURE 2:**
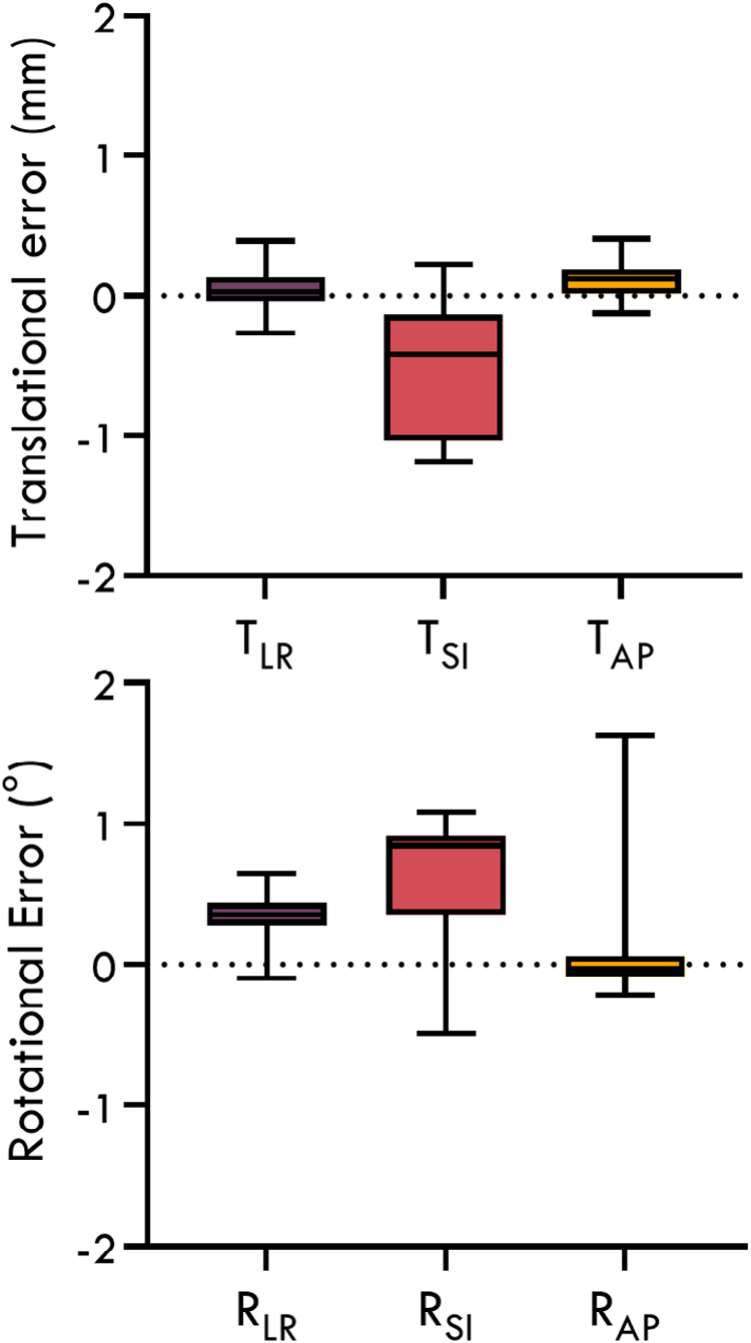
The geometric error of the pelvic tracking motion monitoring method for translation and rotation in the patient coordinate system for 20 patients across five fractions. The whiskers represent the minimum and maximum values.

The overall geometric accuracy of the pelvic motion monitoring method with reference to the patients’ coordinate system is shown in Figure 2. The mean and standard deviation geometric errors for the translational directions $T_{\mathrm{LR}}$, $T_{\mathrm{SI}}$, and $T_{\mathrm{AP}}$ were 0.0 ± 0.1 mm, ‐0.5 ± 0.5 mm, and 0.1 ± 0.1 mm, respectively, and for the rotational directions $R_{\mathrm{LR}}$, $R_{\mathrm{SI}}$, and $R_{\mathrm{AP}}$ were 0.3° ± 0.1°, 0.6° ± 0.4°, and 0.0° ± 0.3, respectively.

The geometric accuracy of prostate motion monitoring using KIM for this cohort of patients has been previously reported to be sub‐mm for translation and within 1.4° for rotation.^23^

### 3.2 6DoF multi‐target displacements

**FIGURE 3:**
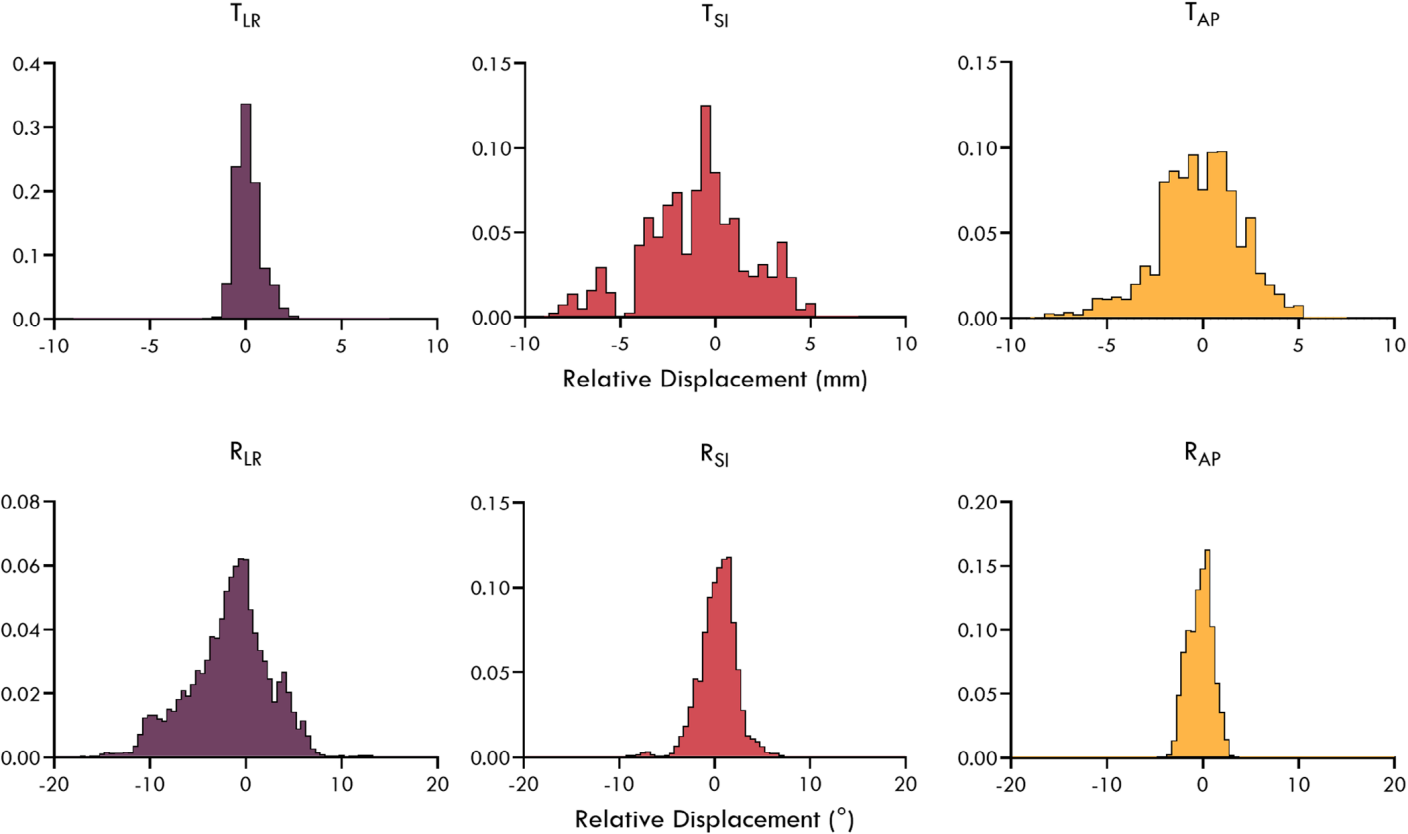
Histograms of the relative translational (top row) and rotational (bottom row) displacements observed between the pelvic bone and prostate across all kV images for the 20 patients analyzed in this study.

**FIGURE 4:**
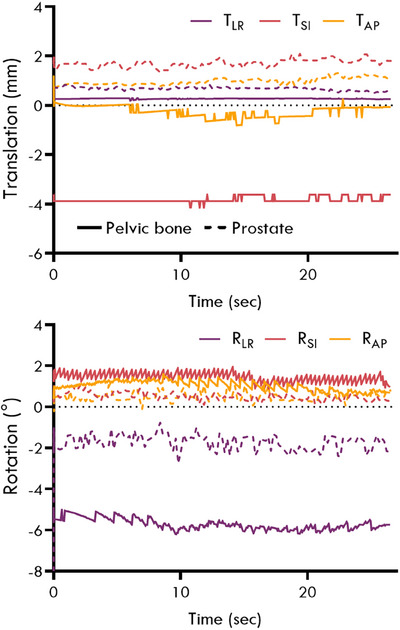
The translation and rotation observed for the pelvic bone (solid lines) and prostate (dashed lines) for one patient. The ground truth displacement for the pelvic bone calculated from the rigid 6DoF registration of the CT to the CBCT was 0.3, ‐2.9, and ‐0.5 mm in the $T_{LR}$, $T_{SI}$, and $T_{AP}$ directions, and ‐6.1°, 0.5°, and 0.5° in the $R_{LR}$, $R_{SI}$, and $R_{AP}$ directions. 6DoF, six‐degrees‐of‐freedom; CBCT, cone‐beam computed tomography.

The distribution of relative displacements between the pelvic bone and prostate observed in the kV projections is shown in Figure 3. The 5th and 95th percentiles for the relative motion between the prostate and pelvic bone were [‐0.8 mm, 1.4 mm], [‐6.2 mm, 3.6 mm], and [‐4.2 mm, 3.2 mm] for the $T_{\mathrm{LR}}$, $T_{\mathrm{SI}}$, and $T_{\mathrm{AP}}$ directions, and [‐9.7°, 4.8°], [‐2.9°, 3.2°], and [‐2.4°, 1.8°] for the $R_{\mathrm{LR}}$, $R_{\mathrm{SI}}$, and $R_{\mathrm{AP}}$ directions, respectively. The 3D relative displacement between the prostate and pelvic bone exceeded 2, 3, 5, and 7 mm for approximately 66%, 44%, 12%, and 7% of the time, respectively. The correlation of motion between the pelvic bone and prostate was small for both translation (*ρ* < 0.3) and rotation (*ρ* < 0.4) in all three directions. An example of the relative motion observed for one patient is shown in Figure 4.

**FIGURE 5:**
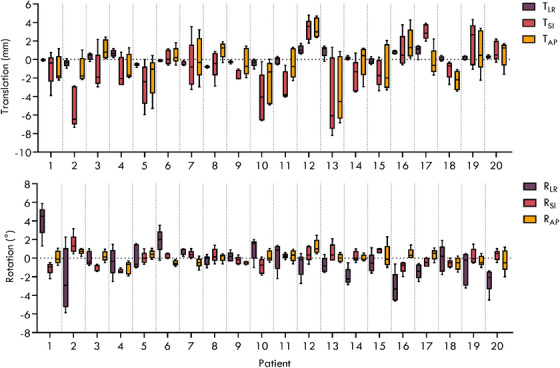
The interfraction translation and rotation of the pelvic bone across five fractions for each patient after the CBCT‐guided couch shift was applied for patient setup. The whiskers represent the minimum and maximum values. CBCT, cone‐beam computed tomography.

After each CBCT scan was acquired during the patient's treatment, a translational couch shift was applied to set the patient up to correctly align the prostate to the isocenter for the beginning of treatment. Rotation was not corrected during patient setup. The resulting interfraction displacements of the pelvic bone for translation and rotation after patient setup for each patient across five fractions are shown in Figure 5.

## 4 DISCUSSION

This study presents an intrafraction pelvic motion monitoring method that would allow for simultaneous motion monitoring of the skeletal anatomy as a surrogate for the pelvic lymph nodes, and the implanted prostate markers using kV images acquired on a standard linear accelerator during treatment. A template matching method that utilized a pre‐generated library of DRRs containing projections of the pelvic bone simulating a range of projection angles to allow for estimation of the pelvic bone translation and rotation was developed and evaluated. The pelvic motion monitoring method was found to have a geometric accuracy and precision within 1 mm and 1° for images acquired for prostate cancer patients treated as part of the TROG 15.01 SPARK trial.

Relative motion between the pelvic bone anatomy and the prostate was also reported. Relative translations between the pelvic bone and prostate were largest in the $T_{\mathrm{SI}}$ and $T_{\mathrm{AP}}$ directions, while rotations were largest around the $R_{\mathrm{LR}}$ axis (pitch). This finding is consistent with previous observations of internal prostate motion.^7^, ^29^, ^30^ The observed relative motions between the pelvic bone and prostate suggest that when motion management is based on the prostate, a 5 mm margin would be sufficient for the pelvic lymph nodes 88% of the time. However, it should be noted that this study was limited to measuring the relative motion between the prostate and pelvic lymph nodes within a short window throughout the acquisition of the pre‐treatment CBCT, and larger displacements could be observed when the entire fraction is considered. Tyagi et al.^31^ similarly examined the relative differences between patient setups based on a fiducial match and bony anatomy match for 30 patients receiving SBRT to the prostate and pelvic lymph nodes. Larger translational shifts were observed in the study by Tyagi et al., where a 5 mm shift would have only covered approximately 75% of patients, and 19% of fractions would have seen a significant loss in dosimetric coverage to the pelvic lymph nodes. Pelvic bone rotations were also measured in the current study, with all pelvic rotations in the $R_{\mathrm{SI}}$ and $R_{\mathrm{AP}}$ directions being within 3°, and rotations in the $R_{\mathrm{LR}}$ direction were within 6°. Larger rotations were seen for the prostate compared to the pelvis and were independent of the pelvic rotations.

An alternative method of estimating 6DoF pelvic bone pose on 2D images has been demonstrated by Munbodh et al.^32^, ^33^ Their registration framework similarly relied on matching pelvic DRRs to a treatment image by finding the pelvic bone position that maximized a similarity measure. However, instead of relying on a library of DRRs with a predetermined set of pelvic rotations, 2D DRRs were computed after iteratively performing rigid spatial transformations of the CT, optimizing for the translation and rotation parameters using a gradient ascent search strategy. While this approach prioritized establishing a high registration accuracy, the computation time to achieve a single registration solution would not be compatible with real‐time intrafraction monitoring of the pelvic bone position. Similar methods relying on fast generation of DRRs have also been applied to verify the 6DoF position of other structures such as the spine^34^ or cranium.^35^ Registration of 2D to 3D images to verify patient setup has also been performed by acquiring orthogonal 2D projections, however the acquisition of images at separate gantry angles on a standard linear accelerator will involve a time delay.^36^ The computational time of the method implemented in MATLAB (MathWorks, USA) in the current study was approximately 10 s per frame. This latency could be reduced considerably for clinical implementation using several strategies including utilizing high performance and parallel computing resources, implementing the method in a faster coding language, reducing the template size, and using gradient ascent search strategies to determine the translational and rotational parameters.

One consideration for the presented method is that the range of pelvic bone rotations that can be estimated is limited by the DRR library that is generated before treatment. In this study, projections with a range of rotations of the pelvis in the $R_{x}$ and $R_{y}$ directions were generated with 0.5° intervals, limiting the precision with which the rotation in these directions could be estimated. In addition, only pelvic rotations in the $R_{x}$ direction ranging from ± 6° were considered in the DRR library, so pelvic rotations larger than this around the $R_{x}$ axis could not be measured in the presented implementation. The search area for consecutive images was also limited to 1 mm and 0.5° which would limit detection of large, abrupt motion. The size of the search window could potentially be modified according to the frequency of image acquisition, such that the search windows are increased for lower image frequencies and decreased for higher imaging frequencies to ensure that any motion occurring between frames is accurately captured. While DRR libraries with a higher angle resolution and a wider range of rotations can be generated to increase the domain of pelvic poses that can be precisely estimated, this would come at the cost of longer DRR generation times and larger memory requirements to load the DRR library at the time of treatment for fast template access. An optimization strategy could also be implemented to speed up the search for the maximum normalized cross‐correlation coefficient to minimize the number of template matches performed. Due to the variations in pelvic bone anatomy between patients, a DRR library will need to be generated for each patient to allow for accurate and precise motion management of bony anatomy.

Integrating pelvic bone motion monitoring with the current KIM method would allow for simultaneous motion monitoring for both the prostate and lymph node targets during radiation therapy treatment. KIM was found to have a geometric accuracy and precision of 0.0 ± 0.4 mm, 0.1 ± 0.3 mm, 0.0 ± 0.5 mm in the $T_{\mathrm{LR}}$, $T_{\mathrm{SI}}$, $T_{\mathrm{AP}}$ directions, and ‐0.1° ± 1.4°, ‐0.1° ± 1.0°, ‐0.1° ± 0.6° in the $R_{\mathrm{LR}}$, $R_{\mathrm{SI}}$, and $R_{\mathrm{AP}}$ directions, respectively for prostate motion monitoring in the TROG 15.01 SPARK trial.^23^ Thus, a combination of the methods would be able to achieve both prostate and pelvic bone motion monitoring to within 0.5 mm and 1.4°. While kV images acquired during treatment delivery may have an increased presence of noise compared to projections acquired during the CBCT due to MV scatter, image quality could be improved through temporal averaging, image filtering, or by acquiring kV images between MV pulses. While patient alignment strategies assume that the pelvic lymph nodes are fixed to the bony anatomy and therefore the pelvic bone is a suitable surrogate for pelvic bone motion,^8^, ^12^, ^37^ magnetic resonance imaging (MRI) studies have indicated that there can be mobility of the lymph nodes relative to the bones with mean absolute deviations of up to 1.1, 3.3, and 2.1 mm in the LR, AP, and SI directions.^38^ Given the low soft tissue contrast in x‐ray images compared to MRI, the pelvic bone position currently provides the best estimate for the pelvic lymph nodes on standard x‐ray guided linacs, but lymph nodes could potentially be tracked more accurately on MR‐linac systems.

Methods to adapt treatment in response to relative multi‐target motion are still limited. While beam gating combined with couch corrections can be used to correct for single‐target motion during treatment, this strategy is not viable for scenarios where multiple targets have undergone differential motion. Online adaptive radiotherapy strategies can be used to account for interfraction displacements that occur between primary targets and the associated lymph nodes by generating a new treatment plan based on the anatomy seen in images acquired on the day of treatment.^12^, ^39^, ^40^ Several studies have seen improvements to the doses delivered to multiple targets simultaneously when modifying the MLC aperture shape and segment weights for intensity‐modulated radiation therapy treatment plans according to relative interfraction shifts.^2^, ^11^, ^41^, ^42^ To adapt to intrafraction motion between multiple targets, real‐time MLC tracking has been demonstrated to adapt the radiation beam to prostate and lymph node targets for patients with locally advanced prostate cancer.^15^, ^43^ As multi‐target MLC tracking can be implemented on standard linear accelerators,^16^ it could potentially be integrated with the multi‐target prostate and pelvic bone KIM motion monitoring proposed in this study to allow for real‐time multi‐target adaptive radiation therapy for locally advanced prostate cancer patients. Applying the real‐time 6DoF bony anatomy targeting method could be investigated for other sites, such as the spine, as the accurate delivery of radiation therapy to the spine is even more crucial. Spine position monitoring has been previously investigated during SBRT delivery, however, has been limited to 3D motion^44^, ^45^ or rotation only in the imaging plane.^46^

Further work into multi‐target motion monitoring could expand into monitoring multiple targets for other anatomical sites. The seminal vesicles are typically included in the target volume for prostate cancer patients, however, deformations resulting in relative motion between the prostate and the seminal vesicles are known to occur.^47^, ^48^, ^49^ This motion is not monitored during treatments with the combined volume instead being treated as being rigid, requiring relatively large PTV margins to be used. Relative displacements of targets are also a known problem for lung^50^, ^51^ and oligometastatic patients.^52^ The increase in availability of combined MR‐linac systems^53^, ^54^ could lead to an improvement in capabilities to simultaneously monitor multiple targets during radiation therapy as well as the motion of nearby organs‐at‐risk to guide further dose‐avoidance during treatment.

## 5 CONCLUSION

This study described a method to allow for the displacement of the pelvic bone to be monitored simultaneously with prostate motion in 6DoF using 2D kV images. The method was retrospectively applied to data acquired during patient treatment from the TROG 15.01 SPARK trial and sub‐mm and sub‐degree geometric accuracy and precision of pelvic bone tracking were demonstrated. The integration of an intrafraction pelvic bone motion monitoring method with prostate tracking could enable image‐guided real‐time multi‐target adaptation to occur during radiation therapy for patients with locally advanced disease.

## CONFLICT OF INTEREST STATEMENT

P.J. Keall and P.R. Poulsen are inventors on a patent related to the KIM technology that is licensed to Varian Medical Systems by Stanford University and are inventors on additional patents/patent applications related to the KIM technology that have been assigned to the SeeTreat. P.J. Keall is the founder and director of SeeTreat.
